# Medial cerebellar nuclear projections and activity patterns link cerebellar output to orofacial and respiratory behavior

**DOI:** 10.3389/fncir.2013.00056

**Published:** 2013-04-02

**Authors:** Lianyi Lu, Ying Cao, Kenichi Tokita, Detlef H. Heck, John D. Boughter Jr.

**Affiliations:** Department of Anatomy and Neurobiology, University of Tennessee Health Science CenterMemphis, TN, USA

**Keywords:** deep cerebellar nucleus, respiration, brainstem, neuronal tract-tracers, orofacial

## Abstract

There is ample evidence that the cerebellum plays an important role in coordinating both respiratory and orofacial movements. However, the pathway by which the cerebellum engages brainstem substrates underlying these movements is not well understood. We used tract-tracing techniques in mice to show that neurons in the medial deep cerebellar nucleus (mDCN) project directly to these putative substrates. Injection of an anterograde tracer into the mDCN produced terminal labeling in the ventromedial medullary reticular formation, which was stronger on the contralateral side. Correspondingly, injection of retrograde tracers into these same areas resulted in robust neuronal cell labeling in the contralateral mDCN. Moreover, injection of two retrograde tracers at different rostral–caudal brainstem levels resulted in a subset of double-labeled cells, indicating that single mDCN neurons collateralize to multiple substrates. Using an awake and behaving recording preparation, we show that spiking activity in mDCN neurons is correlated with respiratory and orofacial behaviors, including whisking and fluid licking. Almost half of the recorded neurons showed activity correlated with more than one behavior, suggesting that these neurons may in fact modulate multiple brainstem substrates. Collectively, these results describe a potential pathway through which the cerebellum could modulate and coordinate respiratory and orofacial behaviors.

## INTRODUCTION

Although it is well established that the cerebellum plays an important role in motor coordination and learning, the neuronal mechanisms underlying cerebellar function are only poorly understood. Historically, cerebellar research has focused mostly on eye movements, posture, and appendicular coordination. However, there is increasing evidence for a role of the cerebellum in the modulation of both respiratory and orofacial movements, the latter including movements such as coughing or whistling in humans, and fluid licking and rhythmic whisking in rodents (Bassal and Bianchi, 1982; Huang et al., 1993; Gozal et al., 1994; Welsh et al., 1995; Xu and Frazier, 1997; Paxinos and Franklin, 2001; Chen et al., 2005; Dresel et al., 2005; Bosman et al., 2010; Bryant et al., 2010; Cao et al., 2012a;Farrell et al., 2012). These movements are generated largely by brainstem pattern generating circuits (e.g., Travers, 2004; Feldman and Del Negro, 2006).

The primary cerebellar output neurons are located in the deep cerebellar nuclei (DCN); these neurons project to a variety of areas, including thalamus, midbrain, and brainstem (Teune et al., 2000). The primary divisions of the DCN (medial or fastigial, interposed, lateral or dentate) can themselves be roughly classified with regards to motor control of body region, with medial DCN (mDCN) neurons chiefly concerned with medial body areas, including trunk, proximal limb, and head (Sugihara, 2011). Neurons in the mDCN have been shown to project bilaterally to at least two sites in the brainstem, the dorsally located vestibular nuclei, as well as to the ventromedial reticular formation (RF). Studies with transgenic mice indicate that contralateral mDCN brainstem projections are excitatory (glutamatergic), whereas ipsilaterally projecting neurons are inhibitory (glycinergic; Bagnall et al., 2009).

There is strong evidence that the cerebellum modulates respiratory-related motor activity via projections of mDCN neurons to various medullary or pontine regions (Lutherer and Williams, 1986; Gruart and Delgado-Garcïa, 1992; Xu and Frazier, 1995, 1997, 2000; Zhang et al., 1999). However, the pathways by which the cerebellum engages brainstem centers controlling other orofacial movements such as licking or whisking is not known. The fact that these movements are widely represented in Purkinje cell activity in the cerebellum (Welsh et al., 1995; Bryant et al., 2010; Cao et al., 2012a) and coordinated with breathing (Welzl and Bures, 1977; Weijnen et al., 1984; Cao et al., 2012b; Deschênes et al., 2012) suggests the possibility of a common pathway with respiratory control from DCN to brainstem.

We investigated this pathway in inbred C57BL/6J (B6) mice by combining neuroanatomical and physiological approaches. Neural tracing studies were performed in order to carefully delineate the projection of mDCN neurons to brainstem locations associated with the generation of respiratory rhythm and mystacial whisker movements. We also recorded single unit activity of mDCN neurons in awake and behaving mice in order to determine whether and how whisker and respiratory behaviors is represented in the activity of these neurons.

## MATERIALS AND METHODS

### ANIMALS

Data were collected from male and female adult C57BL/6J mice (18–30 g). Animals were maintained in standard cages in a temperature- and humidity-controlled vivarium on a 12-h light/12-h dark cycle, and were given *ad libitum* access to normal dry pellet food (22/5 rodent diet, Harlan Teklad, Madison, WI, USA) and water. The Animal Care and Use Committee at University of Tennessee Health Science Center approved this study, and all experiments were carried out in accordance with the National Institute of Health Guide for Care and Use of Laboratory Animals (NIH Publications No. 80-23), revised 1996.

### TRACT-TRACING EXPERIMENTS

#### Surgery and groups

Mice were divided into anterograde and retrograde tracing groups. Prior to surgery for tracer injections mice were anesthetized (i.p. injection) with ketamine/xylazine (100/10 ml/kg) and positioned in a stereotaxic frame (Stoelting, Wood Dale, IL, USA). The scalp was opened with a midline incision, and the skull was leveled between bregma and lambda by adjusting the bite bar. Body temperature was maintained at 35°C using a heating pad. For injections of anterograde neuronal tracer, a glass micropipette filled with 10% biotinylated dextran amine (BDA; 10,000 MW, Invitrogen Corporation, Carlsbad, CA, USA) was lowered into the mDCN using the following coordinates: anteroposterior = 6.24 mm, mediolateral = 0.8 mm, and dorsoventral = -3.3 mm, relative to bregma. For injection of retrograde neuronal tracers, a glass micropipette filled with 5% Fluorogold (FG; Fluorochrome, LLC, Denver, CO, USA) or either red and green latex microspheres (Lumafluor, Durham, NC, USA) was lowered into either rostral ventromedial RF (anteroposterior = -5.88 mm, mediolateral = 0.7 mm, and dorsoventral = -5.75 mm) or caudal ventromedial RF (anteroposterior = -7.08 mm, mediolateral = 1.0 mm, and dorsoventral = -5.85 mm). BDA and FG were injected via iontophoresis (Precision Current Source, Stoelting Co., Wood Dale, IL, USA), at 2 μA (cycle 8 s ON and 8 s OFF), for a total of 10 min. Microspheres were pressure injected (60 nl) via a Picospritzer (Parker Hannifin Corp., Cleveland, OH, USA). The injection pipette was left in place for 10 min before and after the injection was made. Supplemental anesthetic was administered as necessary throughout the surgery to maintain the animals under deep anesthesia.

#### Tissue preparation and imaging

After a 5-day survival period, mice were perfused transcardially with phosphate-buffered saline and 4% paraformaldehyde. The brains were removed and placed in 4% paraformaldehyde for 1 day and then transferred to a 30% buffered sucrose solution and stored at 4°C for at least 1 week. Coronal sections (40 μm, every other section) were cut serially using a freezing microtome.

For visualizing BDA, sections were rinsed, followed by pretreatment with 3% H_2_O_2_ and 0.4% Triton X-100. Sections were then rinsed again and incubated in avidin-biotin complex (ABC) solution prepared with the Vectastain ABC Elite kit (Vector Laboratories, Burlingame, CA, USA). BDA was visualized as a black reaction product using nickel intensification of the chromagen, 3,3′-diaminobenzidine (DAB; Vector). The sections were then rinsed, mounted, air-dried, and coverslipped on silane-coated slides (Scientific Device Laboratory, Des Plaines, IL, USA) with di-*n*-butyl phthalate in xylene (DPX) mountant for histology (Fluka).

The retrograde tracers used in this study could be visualized using fluorescent microscopy, so no further histological processing was necessary. Sections were rinsed, mounted, air-dried, and coverslipped on silane-coated slides with DPX mounting media for histology. All histological material was visualized and imaged using a Leica (DMRXA2, Leica Microsystems, Bannockburn, IL, USA) episcopic-fluorescence microscope equipped with a digital camera (Hamamatsu ORCA-ER, Hamamatsu Corp., Shizuoka, Japan) and imaging software (SimplePCI, Hamamatsu Corp., Shizuoka, Japan).

#### Analysis of histological material

Anterograde tracing: For each mouse (*n* = 6), brainstem/cerebellum sections were examined via light microscopy for BDA labeling, ranging from about 120 μ caudal to the central canal-fourth ventricle border (about -7.6 mm from bregma; Paxinos and Franklin, 2001) to just prior to the appearance of the pontine parabrachial nucleus (about -5.8 mm from bregma). The BDA injection site was found in each case to be confined to the mDCN, centered at about -6.2 to -6.4 mm from bregma, but tending to spread into one or a few adjacent sections in both rostral and caudal directions. Retrograde tracing: All tracer injections (*n* = 15) were made into the medial RF, targeting the areas where terminal labeling was found. There was some variation in the location of the injections, predominantly along the dorsal–ventral axis. The distribution of labeled cells in the DCN was examined using fluorescent microscopy, and fluorescent cell profiles were counted within each subnucleus from three representative sections along the rostral–caudal axis, at approximately -6.6, -6.4, and -6.2 mm from bregma. Cell counts were summed in subnuclei across levels and averaged across mice. Subnuclear labeling was compared between rostral and caudal injection groups, and between ipsilateral and contralateral sides via two-way analysis of variances (ANOVAs). Divisions and nuclei of the brainstem and cerebellum were delineated using both Paxinos and Franklin (2001) and an existing archive of nissl-stained B6 brains in the Authors’ lab. Additional mice were excluded from both the anterograde and retrograde studies due to unsuccessful injections (i.e., one or the other injection not expelled properly, or not on target).

### ELECTROPHYSIOLOGY EXPERIMENTS

#### Surgery

Mice (*n* = 8) were anesthetized initially with 3% isoflurane in oxygen in an incubation chamber. Anesthesia was maintained with 1–2% isoflurane during surgery using an Ohio Isoflurane vaporizer (Highland Medical Equipment). The depth of anesthesia was adjusted until the mice failed to show a withdrawal reflex to a strong pinch of the hindpaw. Rectal temperature was maintained at 37–38°C with a servo-controlled heat blanket (FHC, Inc., Bowdoin, ME, USA). Standard surgical techniques were used to secure three small machine screws in the skull (1/8^′′^ dome head, 0.8 mm diameter, 2 mm long; Small Parts). A craniotomy (2–3 mm diameter) was performed to expose the vermis and part of the right cerebellar hemisphere, leaving the dura intact. A cylindrical plastic chamber was placed over the craniotomy and filled with triple antibiotic ointment to keep the dura moist and reduce infection risk. A custom-made head post was placed in a stereotaxically defined position relative to bregma (Bryant et al., 2009). The chamber and head post were secured to the skull screws with dental acrylic (teets methyl methacrylate denture material; Co-Oral-Ite Manufacturing). A 3- to 4-day postsurgical recovery period was observed before starting electrophysiological experiments. Mice were adapted to the head-fixed situation during two sessions of head fixation of 15 min on the day prior to the first recording.

#### Electrophysiology and behaviors

Access to water in the home cages was restricted 12 h before electrophysiological experiments. All experiments were performed during the light cycle. During experiments, the mouse’s head was fixed to a metal holder and the body was loosely covered with a plastic tube to limit body movements (Bryant et al., 2009). The recoding chamber was cleaned and filled with Ringer’s solution. Up to seven recording electrodes (glass-insulated tungsten/platinum; 80 μm O.D.; impedance, 3–7 MΩ) were inserted into the mDCN using a computer-controlled microdrive (System Eckhorn; Thomas Recording). Neurons in this subnucleus were identified based on their location (bregma -6 to 6.5 mm, 0.5–1 mm lateral from midline, depth 2–2.5 mm) and firing characteristics. The raw signals were bandpass filtered (200 Hz to 8 kHz) and amplified using a filter amplifier (FA32; Multi Channel Systems). Filtered and amplified voltage signals were digitized and stored on a computer hard disk (16 bit A/D converter; sampling rate, >20 kHz) using a CED power1401 and Spike2 software (both Cambridge Electronic Design).

Respiratory behavior was monitored with a thermistor (Measurement Specialties) placed in front of one nostril. Breathing cycles were measured as increases and decreases in temperature caused by exhale and inhale movements, respectively. Raw licking or respiratory signals were digitized at 1 kHz. On the left side of the face all but one whisker (the C4 or C3) was cut. A light-beam sensor (Coulbourn Instruments) was placed beside the mouse’s head at the level of the nose so that the whisker would break the light-beam during large-amplitude protraction movements. The beam-break resulted in a brief bi-phasic potential that was detected off-line to mark the timing of whisker movements using a threshold procedure (Cao et al., 2012b). Licking behavior was monitored with a piezo foil sensor, which created a voltage signal due to deformation at the moment when the mouse’s tongue touched the waterspout. Raw respiratory, whisking and licking signals, and DCN spike activity were recorded simultaneously and stored to the same data file for off-line analysis.

After each experiment, the chamber was rinsed with sterile Ringer’s solution and filled with triple antibiotic ointment. During the last two experimental sessions, small electrolytic lesions (5 μA/10 s) were formed in stereotaxically defined locations using the head post as reference.

#### Electrophysiology data analysis

Single-unit DCN neurons were identified based on sustained spontaneous firing rate (8–50 Hz) and the location of lesions. Peaks and troughs in the air temperature recordings corresponded to the ends of expiration and inspiration cycles, respectively. The end-of-expiration times were marked with a peak-detection algorithm and used as temporal aligns for correlation analyses. The times of whisker protraction movements were detected using a fixed voltage threshold. Analyses of cross-correlations between mDCN neuronal activity and behavior were performed using the Spike2 software (CED, Cambridge, UK). *Z*-scores >2 were considered to be significant. We used the end-of-expiration times as the temporal align for respiratory behavior.

## RESULTS

### NEURAL TRACING

**FIGURE 1:**
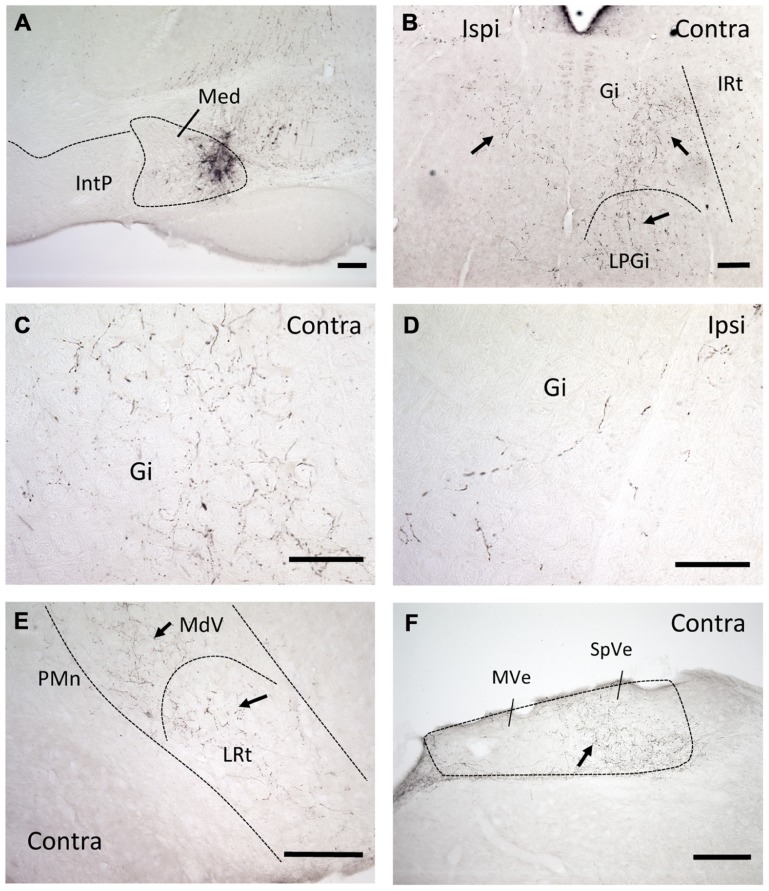
**mDCN neurons project to the contralateral reticular formation, as revealed by anterograde tracing**. **(A)** Tracer injection site in the mDCN (Med), approximately -6.3 from bregma. **(B)** Low power image of labeling in the rostral brainstem in both the ipsilateral and contralateral Gi and LPGi, approximately -6.0 from bregma. At higher power, punctate labeling indicates terminal fields in contralateral Gi **(C)**; relatively fewer terminals are found on the ipsilateral side **(D)**. The level for **(E,F)** is approximately -6.8 from bregma. **(E)** Low power image of labeling in the caudal brainstem, in the contralateral MdV and LRt, approximately -7.3 mm from bregma. **(F)** Labeling in the contralateral vestibular nucleus, approximately -7.0 from bregma. With the exception of **(F)**, all images were prepared from a single mouse brain. Scale bars: **(A,B,E,F)** = 200 μ; **(C,D)** = 100 μ. Arrows point to examples of terminal/axonal labeling in lower power images.

Injections of the anterograde tracer BDA into the mDCN of six B6 mice revealed that neurons in this subnucleus project bilaterally primarily to two areas in the medulla: the complex of vestibular nuclei, located dorsally (MVe and SpVe, respectively), and medial and ventromedial portions of the RF (**Figure 1**). Staining appeared both as punctate varicosities, which appear to be terminal-like arborizations, as well as linear profiles, indicative of axonal labeling. Even though in each case the injection was made at roughly the same rostral–caudal level of the DCN, at least some terminal-like labeling was typically found in the brainstem from the most rostral to the most caudal section examined (range about -5.8 to -7.6 mm from bregma). In the RF labeling was restricted to medial and ventromedial regions, and was stronger on the contralateral side. At more rostral levels, this was predominantly in the gigantocellular nucleus (Gi), but also in the lateral paragigantocellular nucleus (LPGi). Terminal labeling continued caudally in the ventral medullary reticular nucleus (MdV) and medial portion of the lateral reticular nucleus (LRt).

**FIGURE 2:**
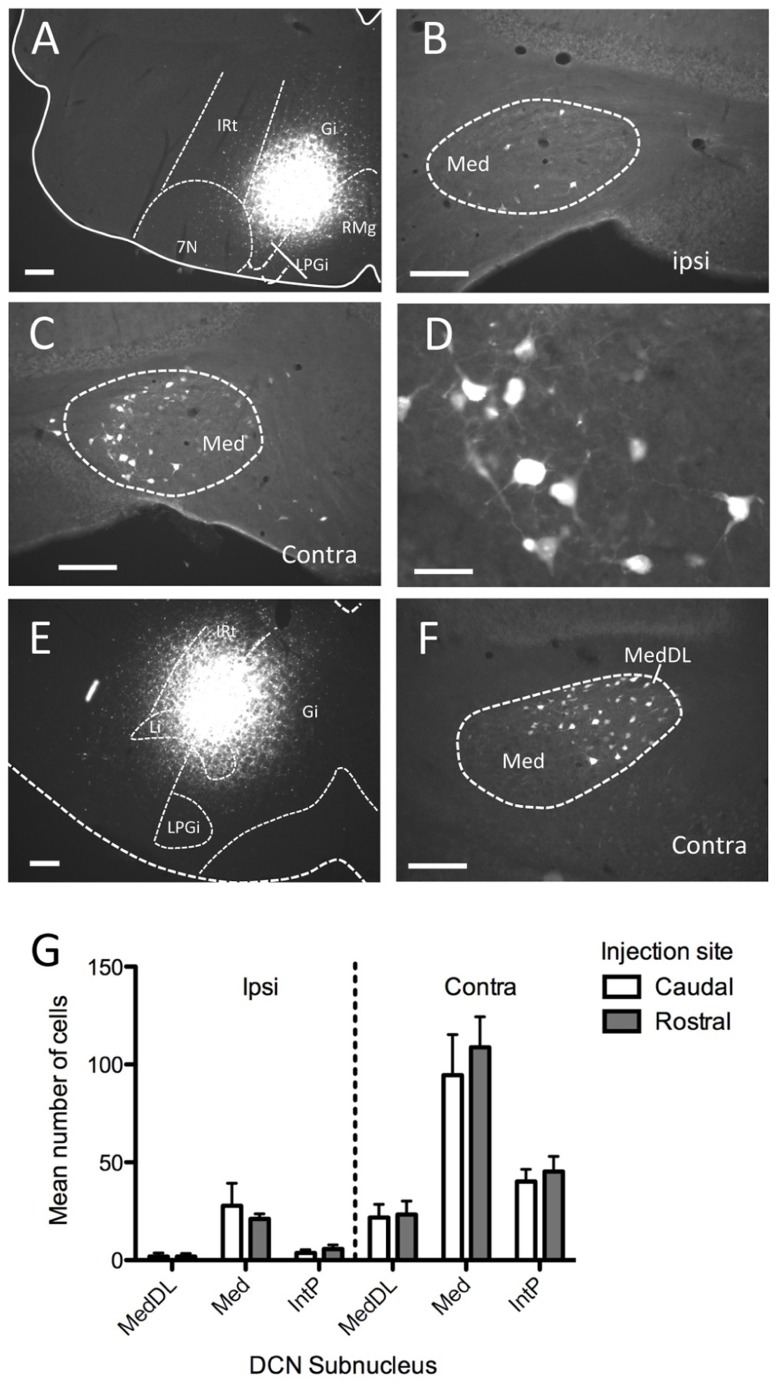
**Injection of the retrograde tracer Fluorogold into ventromedial brainstem labels neurons in the mDCN**. **(A–D)** Following injection into Gi in the rostral medulla **(A)** neurons were sparsely labeled in the ipsilateral mDCN **(B)**, whereas greater numbers of neurons were labeled in the contralateral mDCN **(C)**. The rostral–caudal level for **(B,C)** is approximately -6.2 mm from bregma. **(D)** Higher magnification of retrogradely labeled neurons from **(C)**. **(E,F)** Following injection into the Gi and LPGi at a more caudal level of the medulla **(E)**, neurons are labeled in the contralateral mDCN, including MedDL **(F)**; approximate level is -6.6 mm from bregma). **(G)** Quantification of labeled cells in DCN subnuclei from mice with tracer injections placed caudally (open bars) or rostrally (shaded bars; *n* = 6 per group) reveals no significant difference between the groups. For both groups, significantly more labeled cells were found on the contralateral side (*p* < 0.0001). Scale bars: **(A–C,E,F)** = 200 mm; **(D)** = 50 mm.

We next examined labeling of neuronal cell bodies in the DCN following injection of a retrograde tracer (FG) in the ventromedial RF either at rostral (*n* = 6) or caudal (*n* = 6) levels. These injections were targeted toward the medial RF (Gi) and varied somewhat along the dorsal–ventral axis of the coronal plane, often invading the LRt (caudally) and LPGi (rostrally). In each case, robust neuronal labeling was found in the contralateral mDCN following brainstem injection (**Figure 2**). At caudal levels, the mDCN can be divided into a medial portion (Med) and the dorsolateral hump (MedDL); labeling was often found in both parts. In most cases, at least some labeled neurons were also found in the medial part of the contralateral interposed (IntP) nucleus. Ipsilaterally, fewer labeled neurons were found in any DCN subnucleus, including Med and MedDL [*F*_(1,66)_ = 77.42; *p* < 0.0001]. In the mDCN, many of the FG-labeled neurons tended to be relatively large (20–30 μm along longest axis) and stellate in shape, often with identifiable dendritic or axonal processes (**Figure 2D**). Whether injections were placed at caudal or rostral levels, FG-positive neurons were typically found at all levels of the DCN; moreover, the injection groups did not significantly differ in number of labeled neurons (**Figure 2G**). This equivalency may be in part due to the spread of tracer along the rostral–caudal axis at the injection site. However, it may also reflect potential uptake of FG in mDCN axons passing through the injection site (e.g., Dado et al., 1990).

**FIGURE 3:**
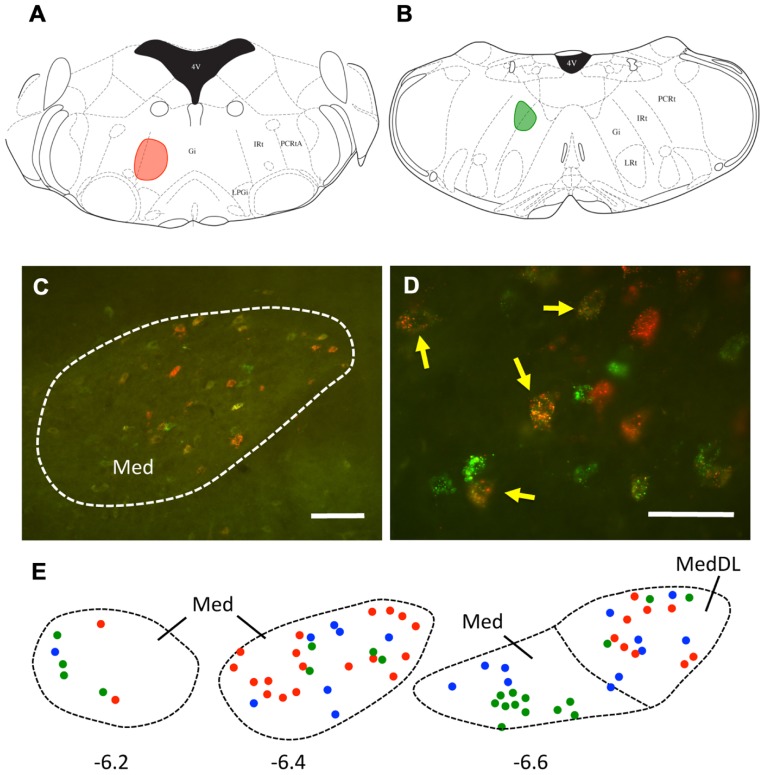
**A subset of mDCN neurons collateralize to rostral and caudal locations in the brainstem**. **(A,B)** Plots of injection sites of rostral (red) and caudal (green) fluorescent microspheres in brainstem. **(C)** Retrogradely labeled mDCN neurons appear green or red, respectively; a subset (yellow) is double-labeled. Labeling is confined to the cell body. **(D)** High power picture of labeling, showing that double-labeled cells (arrows) could be reliably discriminated from single-labeled cells. Approximate level is -6.4 mm from bregma. **(E)** Plots of cells in the mDCN at rostral to caudal levels in a single animal (left to right); a subset of cells at each level are double-labeled (blue circles). Red and green circles correspond to cells labeled by rostral and caudal injections, respectively. Approximate rostral–caudal levels are -6.2, -6.4, and -6.6 mm from bregma (left to right). Scale bars: **(C)** = 100 mm; **(D)** = 50 mm. Atlas sections from Paxinos and Franklin (2001).

It is also possible that individual mDCN neurons may collateralize to both caudal and rostral brainstem levels. To examine this possibility, we injected two different tracers (red and green fluorescent latex microspheres) at both rostral and caudal levels in the same animal (*n* = 3; **Figure 3**; **Table 1**). An advantage of microsphere tracers is that uptake by fibers of passage is minimal (Katz et al., 1984; Apps and Ruigrok, 2007). Injection of microspheres into rostral and caudal levels of the brainstem resulted in a number of double-labeled mDCN neurons in each case, evident at several levels of the mDCN (**Figure 3E**). It is germane to point out that the injections shown in **Figures 3A,B** also varied along the dorsal–ventral axis, with the caudal injection placed more dorsally. This result indicates that a subset of individual cells in this nucleus projects to multiple levels, and potentially different functional areas of the ventromedial RF. As with FG, labeled cells projecting to both brainstem levels co-mingled within the mDCN along its rostral–caudal axis. Overall, fewer retrogradely labeled cells were counted in the mDCN and IntP after microspheres injection relative to FG, likely due to differences in tracer efficacy and injection size. However, the general patterns were similar, and at least some double-labeled cells were found in each subnucleus (16–30% relative to total labeled cells).

**Table 1:** Average number (±SEM) of cells labeled in mDCN and IntP subnuclei from animals in which both rostral and caudal brainstem injections were made (*n* = 3).

Injection group	DCN subnucleus		
	MedDL	Med	IntP
Rostral	17.33 ± 8.69	85.67 ± 31.48	11.67 ± 6.64
Caudal	14.33 ± 6.98	55.00 ± 12.49	3.33 ± 1.76
Double-labeled	8.67 ± 3.84	21.67 ± 4.91	1.67 ± 0.88
% of total that are double-labeled	0.30	0.16	0.04

### RECORDINGS FROM THE DCN

**FIGURE 4:**
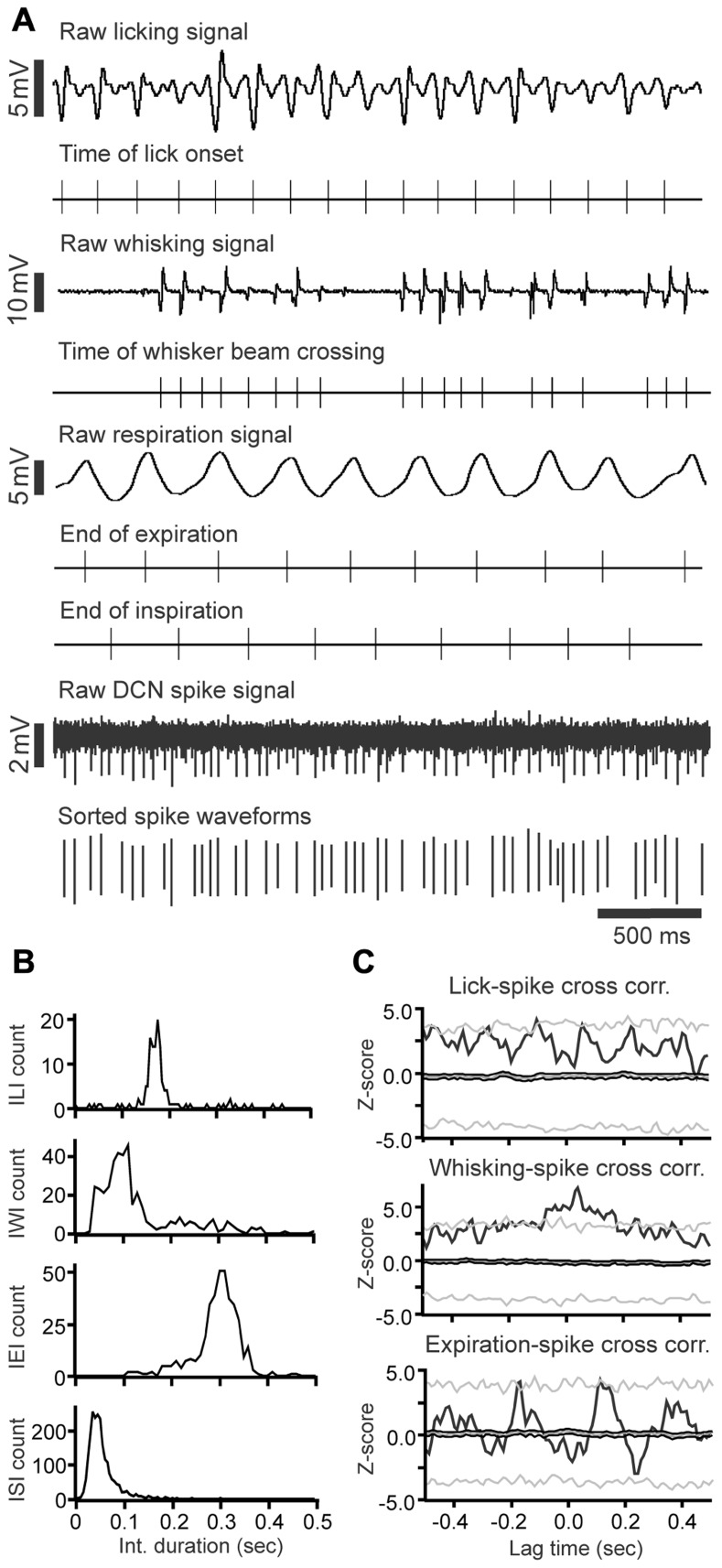
**Raw data example of DCN spike activity correlated with licking, whisking and respiratory behavior**. **(A)** Raw data examples of single-unit DCN spike activity with licking, whisking and respiratory behavior. Under each trace are time markers marking the tongue-to-spout contact times for the licking trace, the time of whisker beam crossing for whisking trace, the end-of-expiration and inspiration times for the respiratory trace, and spike activity for the DCN spike train recording, respectively. **(B)** Histograms showing the inter-lick interval, inter-whisking interval, inter-expiration interval, and inter-spike interval distribution. **(C)** Histograms showing the cross-correlation between DCN spikes with licking, whisking, and expiration events.

**FIGURE 5:**
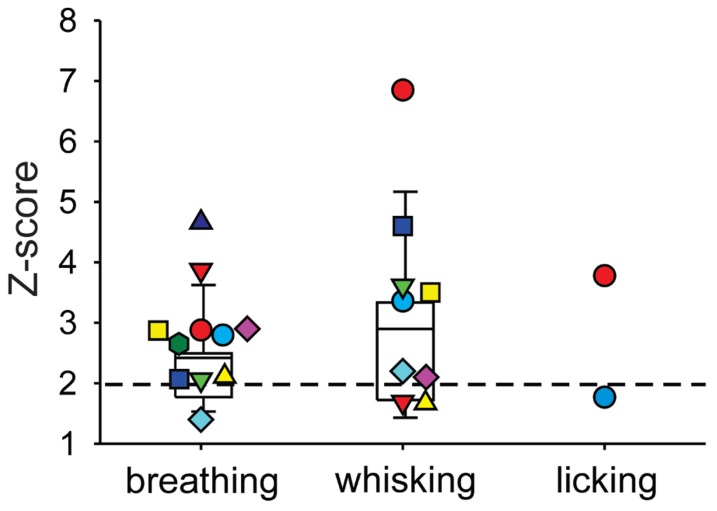
**Summary of behavior-spike cross-correlation results expressed as *Z*-scores of peak correlation values for breathing, whisking, and licking behavior (left, middle, and right column, respectively)**. Each symbol represents one mDCN unit’s *Z*-score for each behavior. Only *Z*-scores > 1 are shown. Spike activity of all 11 units was significantly correlated (*Z*-scores > 2, dashed horizontal line) with either breathing or whisking. Activity of five units was correlated with respiration alone, one unit’s activity was correlated with whisking alone, and the remaining five units had spike activity correlated with both breathing and whisking. One unit (red circle) had spike activity significantly correlated with all three behaviors. Box and whisker plots show median, 25th and 75th percentile (box) and 9th and 91st percentile (whiskers).

We next investigated whether or not DCN single-unit spike activity was related to orofacial and respiratory movements (**Figure 4**). We recorded spike activity from 11 mDCN single units while simultaneously measuring respiration, whisker movements and fluid licking, Cross-correlation analysis was used to determine whether one or more of the three behaviors were represented in the unit’s spiking activity. All 11 mDCN neurons represented either whisking or breathing behavior in their spike firing. As summarized in **Figure 5**, the activity of five neurons was correlated with respiration alone with an average peak correlation *Z*-scores 3.2 ± 1.0 (mean ± SD, *Z*-score range 2.1–4.66), one neuron’s activity was correlated with whisking alone (*Z*-score = 2.2), and the activity of five neurons was correlated with both of these behaviors, with average peak correlation *Z*-scores for breathing of 2.53 ± 0.44 (range 2.06–3.0) and for whisking of 3.44 ± 0.88 (mean ± SD, *Z*-score range 2.12–3.61). Finally, the spike activity of 1 out of 11 mDCN neurons was significantly correlated with all three behaviors with peak correlation *Z*-scores for breathing = 2.88, whisking = 6.85, and licking = 3.78 (red circle in **Figure 5**). Collectively, these physiology data reveal that respiratory and orofacial movements are widely represented in the spike activity of mDCN neurons and that a subset of mDCN neurons represents more than one of these behaviors.

## DISCUSSION

Here we report details of projection patterns of mDCN neurons to the ventromedial RF, a part of the brain stem that contains respiratory and orofacial motor centers. In particular, we found mDCN neurons that send collateral projections to two brain stem sites. Electrophysiological recordings of mDCN neuronal activity in awake behaving mice revealed an extensive neuronal representation of orofacial (licking and whisking) and respiratory movements in the mDCN. All of the 11 mDCN neurons recorded here represented at least one of the three movements in their spike activity. Five out of 11 neurons were multimodal, representing at least two behaviors in their spiking activity. With the caveat that this physiological data is correlative in nature, we suggest that the mDCN ventromedial RF projections described here represent a possible anatomical substrate for a cerebellar involvement in the coordination of brain stem-controlled rhythmic movements.

Both anterograde and retrograde tracing techniques indicate that a subpopulation of neurons preferentially located in the mDCN project diffusely to ventromedial portions of the brainstem RF in mice, including especially the Gi, but also the MdV, LRt, and LPGi. These neurons were distributed in the mDCN along the rostral–caudal axis; this included the MedDL at caudal levels. Projection neurons were also found in the IntP nucleus. Our data confirm results from earlier tracing studies in rats or dogs (Andrezik et al., 1984; Teune et al., 2000). In our experiments, the pattern of labeling was fairly consistent when examined either at rostral or caudal or levels of both the mDCN and brainstem. There was no specific tendency, for example, of rostral mDCN neurons to project to only rostral brainstem. This is consistent with Teune et al. (2000), who found in the rat that injections of an anterograde tracer (either BDA or PhaL) into either caudal or rostral mDCN, as well as into the MedDL, resulted in labeling in the Gi. Injection of retrograde tracers into the Gi revealed that cells that project to the ventromedial RF tended to be fairly large, stellate shaped neurons, similar to large projection mDCN neurons previously described (Bagnall et al., 2009). Further studies with single-cell labeling techniques may yield more precise information on the specific projection topographies of individual mDCN neurons.

### THE mDCN ENGAGES BRAINSTEM SUBSTRATES FOR RESPIRATION AND OROFACIAL MOVEMENTS

A complex grouping of respiratory-related circuits, including pacemaker and premotor neurons, can be found located along the rostral–caudal extent of the brainstem in the ventral respiratory column (rVRC) in the brainstem (Feldman and Del Negro, 2006; Smith et al., 2009). However, the mDCN may not engage these pacemaker areas directly. The rVRC occupies a ventral and lateral position in the medulla and pons (Alheid et al., 2011), a region that did not include terminal labeling from the mDCN in the current study. Moreover, careful examination (via lesion) of certain respiratory sites in the medulla and pons by Zhang et al. (1999) indicate that neurons in a key respiratory rhythm-generating nucleus in the rVRC, the Bötzinger complex, are not necessary for mDCN modulatory effects on respiration. Instead, lesions of the Gi disrupted mDCN-dependent mediation of respiratory timing. Tracing studies suggest that Gi neurons innervate respiratory premotor or motor neurons (Bystrzycka and Nail, 1983; Dobbins and Feldman, 1994), and respiratory-related neurons have been recorded from in this area (Vzaimnykh et al., 1980). Our data confirms that the projection from mDCN to this area of the brainstem is stronger on the contralateral side; Bagnall et al. (2009) posit that that contralateral projections to the ventromedial brainstem are glutamatergic and therefore excitatory, whereas the smaller ipsilateral projections are glycinergic and inhibitory. This reciprocal pattern suggests a mechanism for bilateral coordination.

The location of premotor or rhythmic-generating circuits for other orofacial movements are far less defined than for respiration. Injection of retrograde tracers into the primary motor nuclei for licking and whisking (hypoglossal and facial nuclei, respectively) produce labeling in the RF, including the Gi and MdV, but also the MdD, parvocellular (PCRt) and intermediate (IRt; Travers and Norgren, 1983; Hattox et al., 2002). For licking, evidence suggests a substrate for rhythmic licking organized among premotor neurons in the RF: neurons rhythmically active during licking can be found in both the MdV and dorsal medullary reticular nucleus (MdD) at the level of the hypoglossal nucleus (Travers et al., 1997), as well as in the PCRt, IRt, and Gi divisions, more rostrally. For whisking, serotonergic inputs to vibrissa motor neurons (VMNs) play a key role in the pattern generation, with a necessary role for the motor neurons themselves in rhythmogenesis (Hattox et al., 2003; Cramer et al., 2007). The highest density of serotonergic premotor inputs to VMNs may originate from LPGi neurons; we show here that the LPGi receives input from the mDCN. Although there may be overlap between substrates for licking and whisking there is also likely a degree of rostral–caudal separation, based on proximity to the primary motor nuclei, with the premotor substrate for whisking located more rostrally than that for licking. Respiration, on the other hand, appears to be widely represented in the same areas of the brainstem as licking and whisking. Overall, the specific organization of function within the Gi or other RF areas is not well understood.

### CEREBELLAR ROLE IN THE CONTROL OF RESPIRATORY AND OROFACIAL MOVEMENTS

There is increasing evidence that the cerebellum plays an important role in the modulation of respiration and other types of orofacial movements, such as mastication, licking, swallowing, and whisking (Williams et al., 1986; Welsh et al., 1995; Xu and Frazier, 2000; Suzuki et al., 2003; Bryant et al., 2010). All of these are rhythmic movements controlled by brain stem central pattern generating circuits. It is fairly well established that the cerebellum is involved in the control of respiration, especially with regards to respiratory chemoreception and adaptation of respiratory frequency (Huang et al., 1993; Xu et al., 1994, 2001; Xu and Frazier, 1995, 1997, 2000). Other brain stem-controlled rhythmic orofacial movements with well-documented cerebellar involvement are fluid licking and whisker movements in rodents. Both complex and simple spike activity in Purkinje cells are highly correlated with licking movements in rats and mice (Welsh et al., 1995; Bryant et al., 2010; Cao et al., 2012a). Bosman et al. (2010) demonstrated that Purkinje cell complex and simple spike represent different aspects of mystacial whisker movements in mice. In mice whisking and respiratory movements are correlated in a complex dynamic manner, suggesting that active acquisition of olfactory (sniffing) and tactile (whisking) sensory information are well coordinated rather than independent processes (Cao et al., 2012b). Other orofacial movements such as mastication and swallowing are also coordinated with respiration (Gestreau et al., 2005). Clinical evidence suggests a role for the cerebellum in the control of swallowing movements: patients with cerebellar disease or damage often have difficulties in swallowing (dysphagia; Ramio-Torrentia et al., 2006).

The neuronal mechanisms involved in most of the above cited coordination tasks have thus far received little attention and are only poorly understood. However, the anatomical and electrophysiological findings reported here support a crucial involvement of the cerebellum in the coordination of orofacial and respiratory movements. Our tracing studies suggest that mDCN neurons project to brain stem substrates of orofacial and respiratory movements, and significantly, that individual neurons may project to more than one area, commensurate with the representation of multiple movements in individual mDCN neurons. Collateralization of individual DCN neurons to multiple regions has been noted previously (Bentivoglio and Kuypers, 1982; Bentivoglio and Molinari, 1986). This projection pattern might serve to provide simultaneous cerebellar control of neuronal activity at two or more target sites.

As discussed above, there is increasing evidence for a cerebellar involvement in the coordination of brain stem generated orofacial and respiratory movements. Our electrophysiological and anatomical findings suggest a plausible neuronal substrate for a cerebellar modulation and coordination of brain stem neuronal activity. The anatomical density and complexity of the brain stem circuits poses a major challenge to more detailed investigations of the system. This challenge might best be addressed with evolving techniques of single-cell expression profiling, which have been successfully used genetically identify functionally different classes of neurons (Kodama et al., 2012).

## Conflict of Interest Statement

The authors declare that the research was conducted in the absence of any commercial or financial relationships that could be construed as a potential conflict of interest.
